# Analysis of B Cell Receptor Repertoires Reveals Key Signatures of the Systemic B Cell Response after SARS-CoV-2 Infection

**DOI:** 10.1128/jvi.01600-21

**Published:** 2022-02-23

**Authors:** Yudi Zhang, Qihong Yan, Kun Luo, Ping He, Ruitian Hou, Xinwei Zhao, Qian Wang, Haisu Yi, Huan Liang, Yijun Deng, Fengyu Hu, Feng Li, Xinglong Liu, Ying Feng, Pingchao Li, Linbing Qu, Zhaoming Chen, Qiang Pan-Hammarström, Liqiang Feng, Xuefeng Niu, Ling Chen

**Affiliations:** a Bioland Laboratory, Guangdong Laboratory of Computational Biomedicine, Guangzhou Institutes of Biomedicine and Health, Chinese Academy of Sciences, Guangzhou, China; b State Key Laboratory of Respiratory Disease, Guangzhou Institute of Respiratory Health, the First Affiliated Hospital of Guangzhou Medical University, Guangzhou, China; c Guangzhou Institute of Infectious Disease, Guangzhou Eighth People’s Hospital, Guangzhou Medical University, Guangzhou, China; d University of Chinese Academy of Science, Beijing, China; e Department of Biosciences and Nutrition, Karolinska Institutet, Solna. Sweden; St. Jude Children's Research Hospital

**Keywords:** SARS-CoV-2, antibody repertoire, isotype switching, somatic hypermutation, shared clonotype, B cell receptor

## Abstract

A comprehensive study of the B cell response against SARS-CoV-2 could be significant for understanding the immune response and developing therapeutical antibodies and vaccines. To define the dynamics and characteristics of the antibody repertoire following SARS-CoV-2 infection, we analyzed the mRNA transcripts of immunoglobulin heavy chain (IgH) repertoires of 24 peripheral blood samples collected between 3 and 111 days after symptom onset from 10 COVID-19 patients. Massive clonal expansion of naive B cells with limited somatic hypermutation (SHM) was observed in the second week after symptom onset. The proportion of low-SHM IgG clones strongly correlated with spike-specific IgG antibody titers, highlighting the significant activation of naive B cells in response to a novel virus infection. The antibody isotype switching landscape showed a transient IgA surge in the first week after symptom onset, followed by a sustained IgG elevation that lasted for at least 3 months. SARS-CoV-2 infection elicited poly-germ line reactive antibody responses. Interestingly, 17 different IGHV germ line genes recombined with IGHJ6 showed significant clonal expansion. By comparing the IgH repertoires that we sequenced with the 774 reported SARS-CoV-2–reactive monoclonal antibodies (MAbs), 13 shared spike-specific IgH clusters were found. These shared spike-specific IgH clusters are derived from the same lineage of several recently published neutralizing MAbs, including CC12.1, CC12.3, C102, REGN10977, and 4A8. Furthermore, identical spike-specific IgH sequences were found in different COVID-19 patients, suggesting a highly convergent antibody response to SARS-CoV-2. Our analysis based on sequencing antibody repertoires from different individuals revealed key signatures of the systemic B cell response induced by SARS-CoV-2 infection.

**IMPORTANCE** Although the canonical delineation of serum antibody responses following SARS-CoV-2 infection has been well established, the dynamics of antibody repertoire at the mRNA transcriptional level has not been well understood, especially the correlation between serum antibody titers and the antibody mRNA transcripts. In this study, we analyzed the IgH transcripts and characterized the B cell clonal expansion and differentiation, isotype switching, and somatic hypermutation in COVID-19 patients. This study provided insights at the repertoire level for the B cell response after SARS-CoV-2 infection.

## INTRODUCTION

The outbreak of COVID-19 caused by severe acute respiratory syndrome coronavirus 2 (SARS-CoV-2) presents a great threat to the current global public health due to its rapid transmission and high mortality rates (1, 2). Although suppression of the host immune response was found in the early cases, infection of SARS-CoV-2 will induce the activation of B and T cells, and inflammatory cytokines during the acute infection. There are rapid SARS-CoV-2 nucleocapsid protein (N)- and spike protein (S)-specific antibody responses in both mild and severe cases in the first week after infection (3). The robust B cell responses have accelerated the identification of multiple monoclonal antibodies from patients (4–6), and the development of vaccines to combat the SARS-CoV-2 pandemic (7).

The development of B cells includes the recombination of immunoglobulin (Ig) genes to form naive B cell receptor (BCR) repertoire followed by the elimination of self-reactive B cells in the bone marrow (8). After antigen stimulation, B cells undergo isotype switching from IgM to IgA/IgE/IgG (9, 10). In the germinal centers, the variable (V) genes of antibodies experience somatic hypermutation (SHM) to diversify and enhance BCR affinity and specificity (11). Isotype switching events have been identified based on the analysis of the BCR repertoire of autoimmune disease and HIV-1 infection (12, 13), but a detailed isotype switching profile of SARS-CoV-2 patients remains unknown. Another important aspect of repertoire studies is the search for shared clusters between different individuals after viral infection. Convergent antibody evolution has been found in Ebola virus-infected survivors (14), Ebola vaccine receivers (15), and chronic HIV-1 patients (16). The dynamic landscape of immune repertoires could be visualized by longitudinal analysis of B cell samples from the same patient (17, 18). Furthermore, combined repertoire sequencing with antigen-specific single-cell sequencing could identify virus-specific antibodies (19, 20). Therefore, analysis of antibody repertoires can provide information on the characteristics of the humoral response and facilitate the identification of neutralizing antibodies after infection or vaccination.

Although many studies have characterized the kinetics of serum antibody titers following SARS-CoV-2 infection, B cell clonal expansion and differentiation, isotype switching, and SHM rates remain undefined. We and others recently studied the BCR and T cell receptor repertoire of COVID-19 patients and found that the percentage of TCR-beta could be a signature for disease recovery. In addition, there is a transient IgA surge in the early infection (18, 21). In the present study, we sequenced BCR repertoires of 24 blood samples collected from day 3 to 3 months after SARS-CoV-2 infection. We characterized clonal expansion, clonal differentiation, antibody isotype switching, and somatic hypermutation. We also attempted to identify the shared cluster clonotypes that may be present among COVID-19 patients.

## RESULTS

### Study design and bioinformatics analysis pipeline of IgH repertoires in COVID-19 patients.

**FIG 1:**
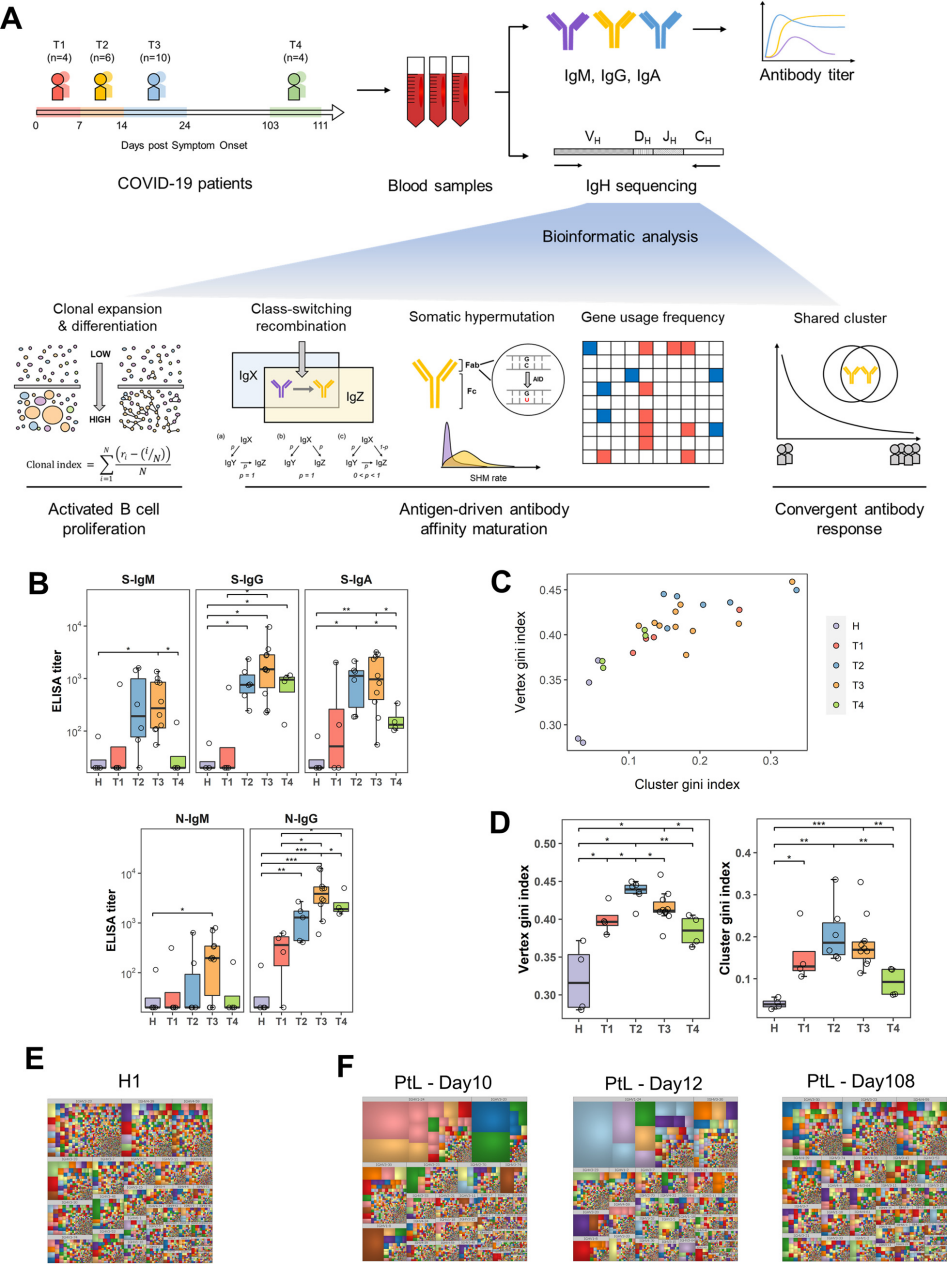
Analysis of antibody response to SARS-CoV-2 infection. (A) Pipeline for antibody repertoire analysis. The process contains serological analysis with serum samples as well as bioinformatic analysis of high-throughput sequencing. (B) Kinetics of S-specific and N-specific IgM, IgG, and IgA antibody ELISA titers of 24 blood samples from 10 COVID-19 patients. T1 (week 1), 1-7 days after symptom onset (*n* = 4); T2 (week 2), 8–14 days after symptom onset (*n* = 6); T3 (week 3), 15–24 days after symptom onset (*n* = 10), in which all patients were SARS-CoV-2 negative; T4, 103-111 days after symptom onset (*n* = 4). Plasma samples from healthy individuals (H) were used for comparison (*n* = 4). *P* values were calculated by *t* test; *, *P* < 0.05; **, *P* < 0.01; ***, *P* < 0.001. (C) Vertex Gini index plotted against cluster Gini index for 24 COVID-19 patient samples and 4 healthy individual samples. (D) Vertex Gini index and cluster Gini index of global IgH repertoires in different phases. (E–F) Treemap for top 10,000 IgH clones in a representative healthy donor (H1) and a representative COVID-19 patient (PtL) on day 10, day 12, and day 108 after symptom onset. For treemap, each rectangle represents a unique V-J-CDR3 sequence. The size of each rectangle reflects the relative proportion of a unique V-J-CDR3 sequence in the treemap. The color of each rectangle was randomly assigned rather than matching to each unique sequence. Each plot showed the top 10,000 most frequent V-J-CDR3 sequences of each IgH repertoire.

To take an overview of the B cell responses induced by SARS-CoV-2 infection, a total of 24 peripheral blood mononuclear cell (PBMC) samples containing about 2,000,000 cells each were collected from 10 COVID-19 patients between 3 days and 3 months after symptom onset (Fig. 1A; Table 1). Longitudinal PBMC samples from eight patients were collected, covering at least two different time points. Four patients (PtL, PtZ, PtK, and PtS) were followed for 10 to 111 days after symptom onset to investigate the dynamics of antibody development against SARS-CoV-2. All of the patients showed mild or moderate symptoms, with an average hospitalization time of 19 ± 4 days (Table 1). Based on the time course of the disease, we divided these samples into four time points: T1 (week 1), 1–7 days after symptom onset (*n* = 4); T2 (week 2), 8–14 days after symptom onset (*n* = 6); T3 (week 3), 15–24 days after symptom onset (*n* = 10), in which all of the patients were SARS-CoV-2 negative, and the samples were collected before discharge from hospital; T4 (near month 4), and 103–111 days after symptom onset (*n* = 4), when the patients were revisiting the hospital. Blood samples from four healthy individuals were also included in this study for comparison (Table 1).

**TABLE 1:** Demographics and characteristics of patients infected with SARS-CoV-2

Donor	Age	Gender	Days of hospital stay	Days post symptom onset	No. of filtered reads	No. of clonotypes	No. of unique clonotypes
PtQ	59	Female	25	3	2,958,647	177,320	4,093,950
				5	3,520,710	235,681	
PtP	34	Male	17	5	3,461,811	231,672	
				14	3,604,579	284,486	
PtF	33	Female	24	7	3,851,775	141,569	
				14	4,423,777	191,436	
				19	5,253,040	243,644	
				22	4,017,839	180,847	
PtL	38	Female	10	10	3,001,974	105,857	
				12	3,064,636	126,992	
				108	3,756,174	245,183	
PtZ	70	Male	16	10	3,245,689	139,437	
				19	3,385,858	244,927	
				108	4,586,379	300,163	
PtG	67	Male	17	13	3,560,827	147,388	
				15	3,396,033	221,269	
				20	3,548,310	342,194	
PtW	59	Female	21	16	4,235,101	301,947	
				21	3,263,512	165,967	
				24	4,446,688	345,782	
PtK	51	Female	23	17	1,675,653	44,648	
				111	3,369,465	292,884	
PtH	53	Male	23	15	3,090,228	171,743	
PtS	68	Female	18	103	3,266,776	330,103	
H1	58	Male	/	/	2,971,932	97,862	1,621,485
H2	28	Male	/	/	3,300,640	122,146	
H3	32	Female	/	/	3,263,761	209,286	
H4	28	Male	/	/	3,618,997	144,998	

The mRNA transcripts of antibody IgH repertoire were amplified using unbiased arm-PCR and sequenced. There were 99.14 million raw IgH sequences considering all of the samples. On average, 3.54 ± 0.12 (mean ± SE) million IgH sequences were obtained for each sample. After pipeline processing, we obtained 4.82 million unique clones for all 10 COVID-19 patients and a total of 1.62 million unique clones across four healthy donors (Table 1). Five principal bioinformatic analyses were performed on the IgH repertoire to identify key signatures in SARS-CoV-2 infected patients: (1) clonal expansion and diversification; (2) antibody isotype switching; (3) somatic hypermutation (SHM); (4) IGHV/IGHJ gene usage and heavy chain complementarity determining region 3 (HCDR3) length; and (5) identification of shared SARS-CoV-2-specific clusters, which use the same inferred V and J genes with over 80% identity in HCDR3 and present in at least three different COVID-19 patients (Fig. 1A).

### SARS-CoV-2 infection induced rapid antibody clonal expansion and diversification.

We first determined the antibodies against SARS-CoV-2 spike protein (S) and nucleocapsid protein (N) using enzyme-linked immunosorbent assay (ELISA). As we reported previously (3), S-specific and N-specific IgG and IgM antibodies were detectable as early as the first week after symptom onset in some patients. IgM and IgG antibody titers continued to increase in the T2 and T3 time points, with all of the patients showing S-specific and N-specific IgG positive in T2 and T3. IgM antibodies showed a sharp decline in the T4 time point back to the level of healthy donors. In contrast, IgG antibodies remained at a relatively high level by 3 months after hospital discharge, consistent with recent findings that S-specific IgG antibodies declined but maintained a stable level for at least 8 months (22, 23). S-specific IgA antibodies had a similar trend as S-specific IgM in T1–T3 and decreased about 50% by the T4 time point (Fig. 1B).

**FIG 2:**
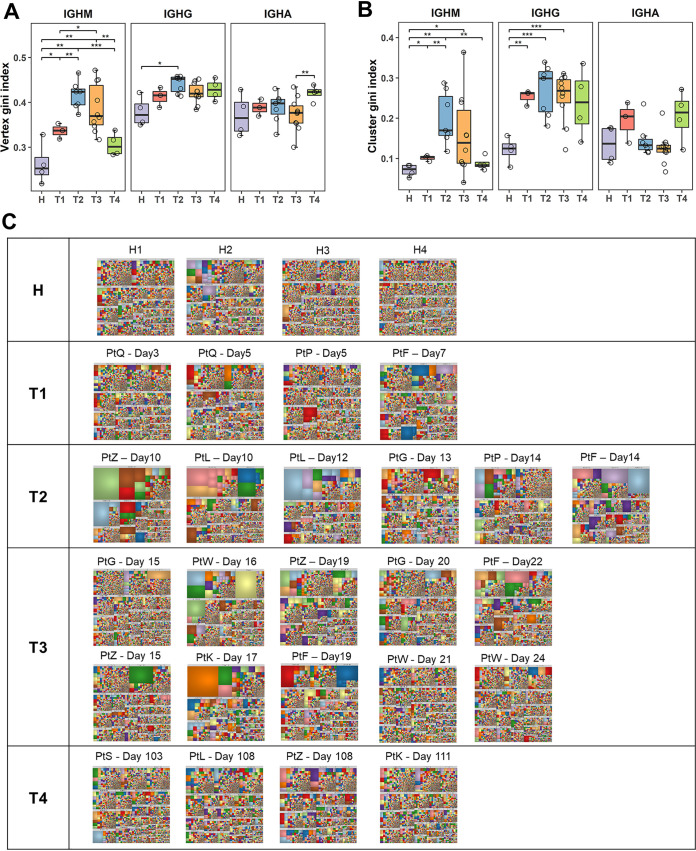
B cell clonal expansion and differentiation detailed to global IgH repertoires and different isotypes. (A–B) Vertex Gini index and cluster Gini index of each isotype in different phases. *P* values were calculated by *t* test; *, *P* < 0.05; **, *P* < 0.01; ***, *P* < 0.001. (C) Treemaps for all IgH repertoires sequenced in this study. Treemaps for healthy individuals and COVID-19 patients with different sampling time points. Each rectangle represents a unique V-J-CDR3 sequence. The size of each rectangle reflects the relative proportion of a unique V-J-CDR3 sequence in treemap. The color of each rectangle was randomly assigned rather than matching to each unique sequence. Each plot shows the top 10,000 most frequent V-J-CDR3 sequences of each IgH repertoire.

To characterize the clonal signatures of different phases in the IgH repertoires of COVID-19 patients and healthy individuals, we calculated the vertex Gini index and cluster Gini index of IgH repertoires as previously described (12, 24). These two indices measure the unevenness of clone and cluster size distribution in the IGH repertoire and reflect the clonal expansion and diversification of B cells (Fig. 1A). We found that the two indexes of the COVID-19 patients in the infection course (T1–T3) and healthy individuals (H) have a distinct distribution. At 3 months after recovery (T4), the index of COVID-19 patients gradually approaches the index of healthy donors (Fig. 1C). The IgH repertoires of COVID-19 patients showed increased clonal expansion and diversification during the infection course (T1–T3), with a peak at T2 (Fig. 1D), demonstrating an active B cell response during the second week after symptom onset. Three months after hospital discharge (T4), such clonal expansion and diversification showed a significant decrease but was still higher than in uninfected healthy people, indicating that the peripheral B cell response was *en route* to a quiescent state (Fig. 1D). Furthermore, the increase of clonal expansion in the repertoires is mostly due to the expansion of naive IgM^+^ B cells. In contrast, the elevated clonal diversification is mainly due to the diversity of IgG^+^ B cells (Fig. 2A and B).

We used treemaps to illustrate the IgH clonal distribution in the repertoire of COVID-19 patients as reported previously in analyzing a Zika virus-infected patient (17). The IgH repertoires of all four healthy donors comprised evenly distributed and no dominant antibody clones, demonstrating that the B cell repertoires are in a relative steady-state equilibrium (Fig. 1E; Fig. 2C). In contrast, the IgH repertoires showed a skewed distribution with increased large or dominant clones in all COVID-19 patients after symptom onset (Fig. 1F; Fig. 2C). Furthermore, these large or dominant IgH clones can be observed in samples collected as early as 7 days after symptom onset, suggesting a rapid activation and clonal expansion of activated B cells. Notably, most large or dominant clones disappeared in T4 (3 months after hospital discharge), indicating that the activated B cells have retreated from their clonal expansion, as the viruses have been cleared from the body and the patients recovered from the disease (Fig. 2C). Therefore, the B cells showed a rapid increase of clonal expansion and clonal diversification in responding to SARS-CoV-2 infection.

### IgH repertoires showed a significant isotype switching from IgM to IgG and a transient increase of IgA after SARS-CoV-2 infection.

**FIG 3:**
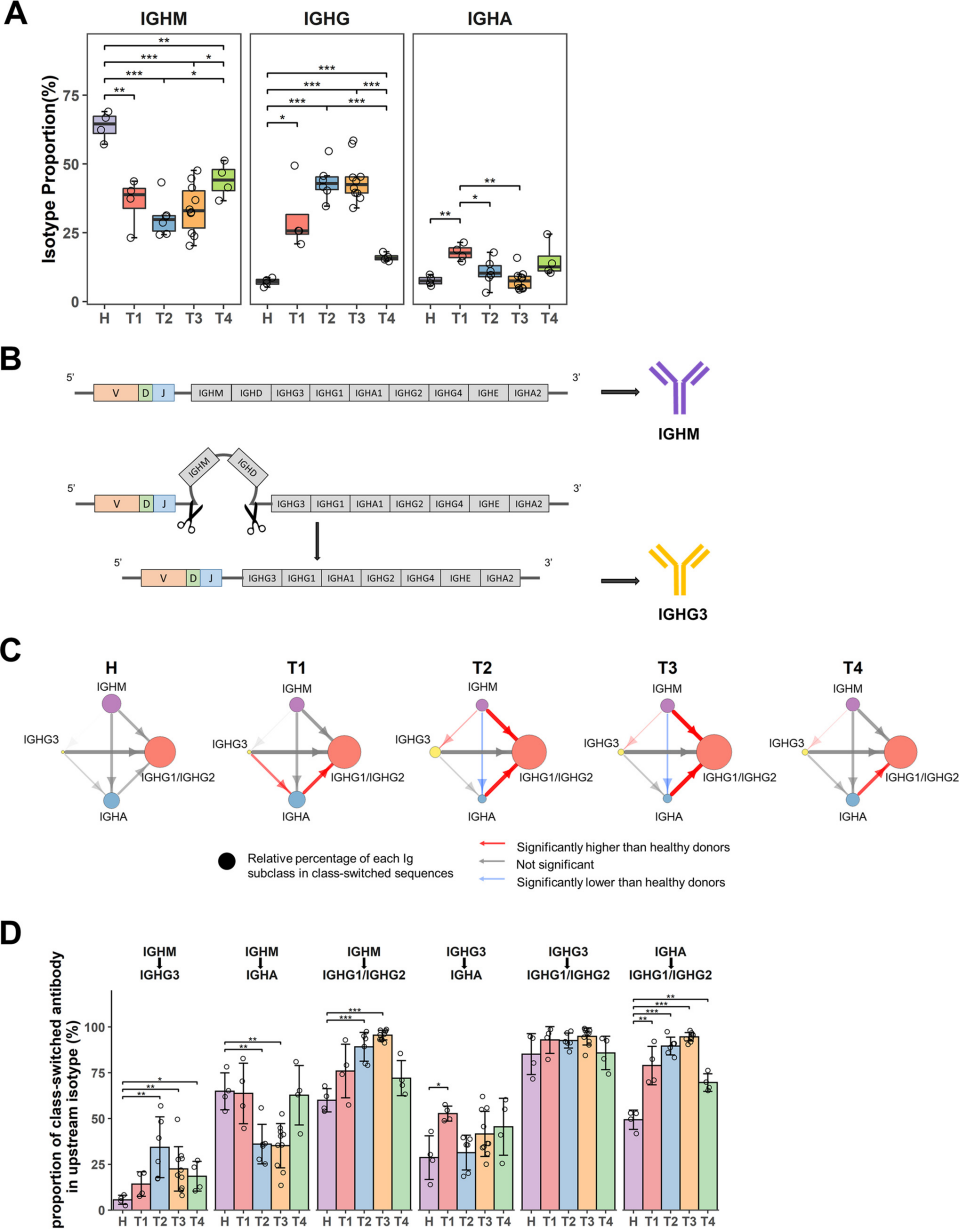
SARS-CoV-2 infection altered isotype proportions and class switching patterns. (A) Proportion of each isotype in different phases of SARS-CoV-2 infection and healthy donors. (B) IgH chain consists of variable region (V, D, J genes) and constant region (C genes). Different Ig subclasses are encoded by corresponding C genes. CSR happens in constant region with specific C gene segments looping out. (C) CSR patterns for COVID-19 patients in each disease phase. Each circle represents an Ig subclass; the size of circle reflects the relative percentage of each Ig subclass in all class-switched sequences; arrow represents the direction of class switching; the thickness of arrow is determined by the proportion of class-switched sequences in all sequences of an Ig subclass; and colors of arrows indicate that the proportion is significantly higher (red), significantly lower (blue), or not significantly changed (black) compared to healthy individuals. (D) Proportion of class-switched antibodies. Bars represent the proportion of class-switched antibody occupying in upstream isotype. *P* value was calculated by *t* test; *, *P* < 0.05; **, *P* < 0.01; ***, *P* < 0.001. *P* < 0.05 was used to determine the significance for class switching.

We next investigated the patterns of antibody isotype distribution in COVID-19 patients. IgM is abundantly expressed, nearly ∼60% in the antibody repertoires of healthy donors (Fig. 3A). In response to SARS-CoV-2 infection, IgG transcripts steadily increased over time, whereas IgM decreased throughout T1–T3 but regained in T4 (Fig. 3A). Notably, there was a transient increase of IgA transcripts in the first week post symptom onset (T1), but decreased subsequently. The relative IgG and IgA abundance coincided with a reduction of relative IgM abundance, indicating that the naive B cells undergo isotype switching and clonal expansion of switched IgG and IgA repertoires (Fig. 3A).

To further characterize the isotype switching or class-switching recombination (CSR) profile in COVID-19 patients, we assessed the progression of CSR between any two Ig isotypes by quantifying the frequency of unique clonotypes shared by two isotypes (Fig. 1A). Based on the order of the IgH constant region loci on the chromosome, isotype switches are irreversible and must proceed from upstream isotypes of IGHM to downstream isotypes, IGHG, IGHA, and IGHE, while the variable VDJ gene remains the same in this progress (Fig. 3B). We devised an algorithm that counts switches between isotypes and calculates the relative frequency of switching between every pair of isotypes (Fig. 1A). We first determined a baseline using the frequency of CSR events in healthy individuals (Fig. 3C). In healthy donors, IgM has a low level of direct switch to IgG1/IgG2 or IgA1/IgA2. Indirect switching (also referred to as sequential switching) pathways from IgG3 to downstream isotypes (IgG1/IgG2, IgA1/IgA2) is rare (Fig. 3C). CSR event differences between isotypes usually corresponded to the dynamic changes in isotype proportion (Fig. 3A, C, and D). Compared with healthy donors, the representation of IgA1 and IgA2 transcripts dramatically increased. Because the sequencing result could not distinguish IgA1 and IgA2 transcripts, we used IgA1/IgA2 to represent total IgA, although the switch regions of IgA1 and IgA2 are located upstream and downstream of IgG2, respectively. The switching from IgG3 to IgA1/IgA2 was significantly enhanced in T1 following infection. In parallel with the observation of elevated IgG transcripts, we found a continuous increase of switches from IgM to IgG1/IgG2 or from IgA1 to IgG2. Notably, the switch from IgA1 to IgG2 was still active in T4, indicating that antibodies continue to have isotype switching even 3 months after acute infection (Fig. 3C and D). Therefore, this systematic analysis of CSR events in COVID-19 patients illustrates disease-specific isotype profiles, revealing a transient increase of IgA transcripts and a steady increase of IgG transcripts in the repertoires.

### Naïve B cells were expanded with low SHM in response to SARS-CoV-2 infection.

**FIG 4:**
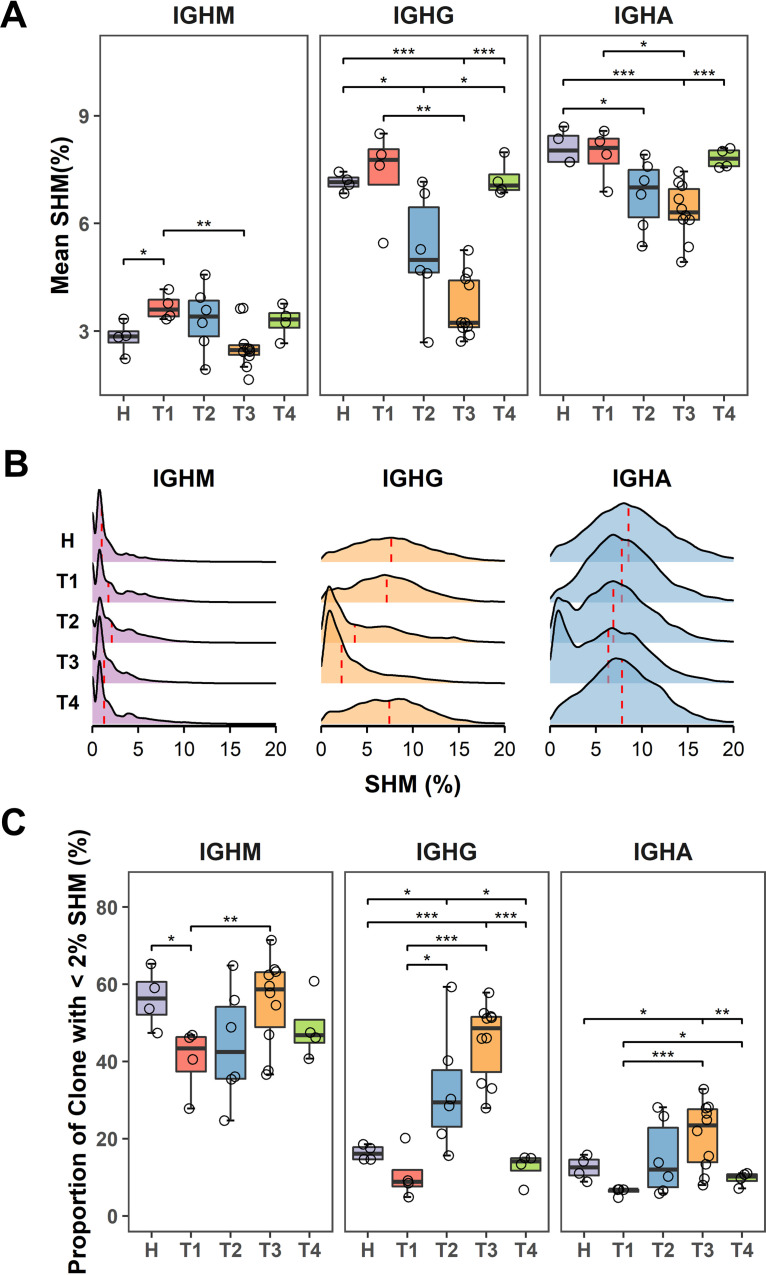
Analysis of somatic hypermutation (SHM) rates in IgH repertoires. (A) Mean SHM rates of IgH repertoire for each isotype were compared among different disease phases. (B) Density distribution of SHM rates for each isotype in different disease phases. Height of peak indicates frequency for a given SHM rate. The vertical red-dotted lines represent median SHM rate. (C) Proportion of low-SHM (< 2%) clones for each isotype in different phases. *P* values were calculated by *t* test; *, *P* < 0.05; **, *P* < 0.01; ***, *P* < 0.001.

SHM introduces point mutations in the antibody variable region that encodes the antigen-binding sites, thus promoting affinity maturation. In healthy donors, IgM is usually expressed by naive B cells with low SHM, whereas the switched isotype IgG or IgA showed higher SHM (IgM: 2.83 ± 0.23%, IgG: 7.24 ± 0.07%, IgA: 8.37 ± 0.23%). Interestingly, COVID-19 patients exhibited significantly lower SHM than healthy individuals (Fig. 4A). The mean SHM of IgM transcripts increased in T1 and T2, then the mean SHM dropped in T3 and recovered to normal level at T4 (Fig. 4A). The mean SHM of total IgG transcripts was unchanged in T1, but became significantly lower in T2 and T3, and then recovered to the average level at T4. The mean SHM of total IgA transcripts also dropped in T2 and T3 time points, and recovered to the normal level at the T4 time point (Fig. 4A). We used a Ridgeline plot to visualize the distribution of IgH transcripts with different SHM. It revealed a remarkable skewing of the SHMs distribution triggered by SARS-CoV-2 infection, with distinct dynamic patterns across different isotypes (Fig. 4B). Bursts of IgG clones with low SHM in T2 and T3 were observed, indicating SARS-CoV-2-specific IgG clones derived from naive B cells were expanded in T2 and T3. Although to a lesser extent, IgA clones showed a trend similar to IgG (Fig. 4B).

**FIG 5:**
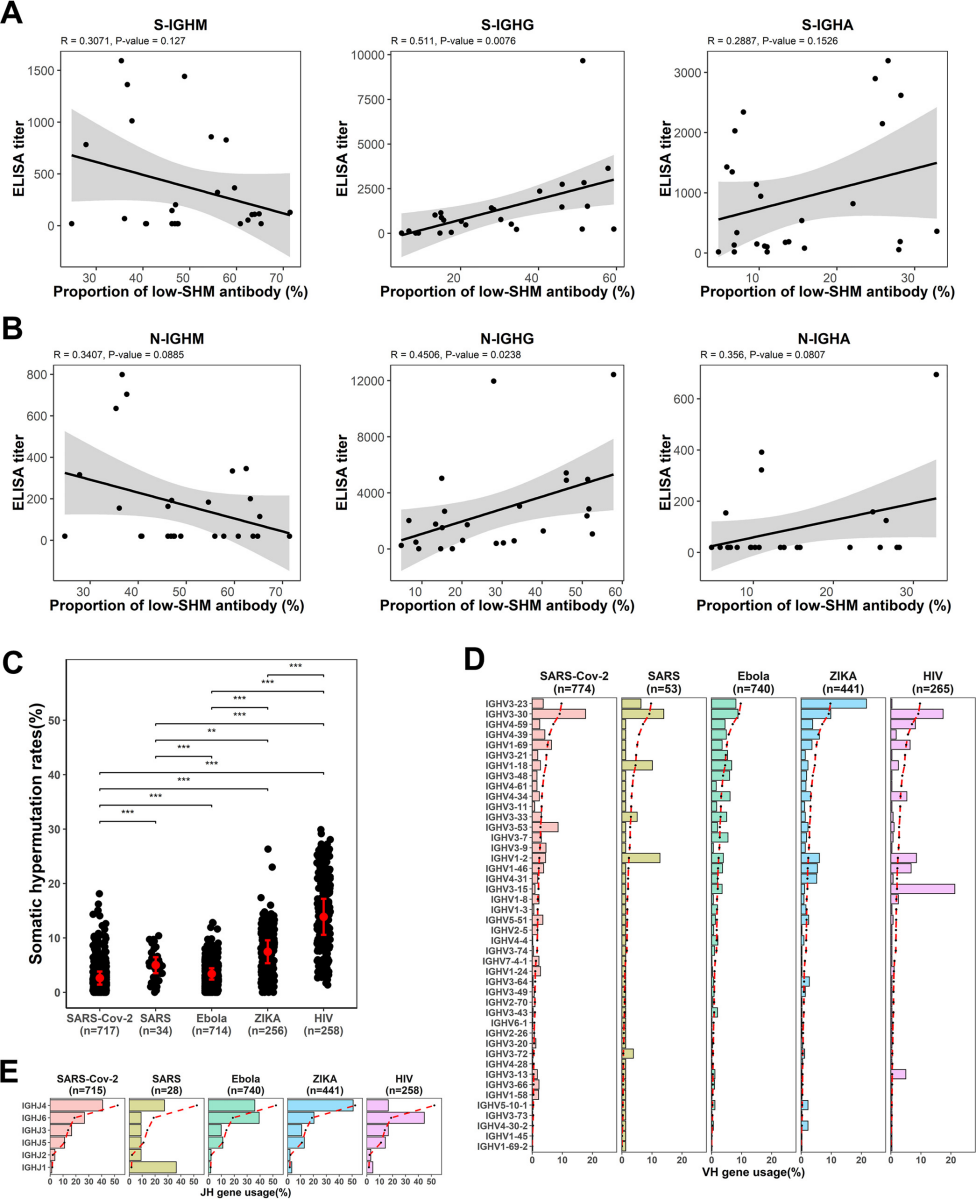
SHM rates and germ line gene usage. (A–B) Correlation between proportion of low-SHM antibody (SHM rate < 2%) and ELISA titer for IgM, IgG, and IgA binding to SARS-CoV-2 spike protein (A) and SARS-CoV-2 nucleocapsid protein (B). (C) Somatic hypermutation rates of specific MAbs for SARS-CoV-2, SARS-CoV, Ebola virus, Zika virus, and HIV were compared. (D–E) IGHV gene usage (D) and IGHJ gene usage (E) of specific MAbs for SARS-CoV-2, SARS-CoV, Ebola virus, Zika virus, and HIV were compared. Dashed lines represent the gene usage of VH and JH in healthy individuals. *P* values were calculated by *t* test; *, *P* < 0.05; **, *P* < 0.01; ***, *P* < 0.001.

We next defined the low-SHM clones with <2% as a cutoff. The proportion of IgG and IgA low-SHM clones steadily increased during T1 to T3 (Fig. 4C). Notably, the low-SHM IgG expressing B cells increased rapidly over time, from 10.67 ± 3.30% in T1 to 32.55 ± 6.35% in T2 and 45.17 ± 3.15% in T3 (Fig. 4C). Furthermore, we found a strong correlation between the proportion of low-SHM IgG sequences and the antibody titers of S-IgG and N-IgG (Fig. 5A and B). Overall, the skew toward low SHM in COVID-19 patients indicates that SARS-CoV-2 infection massively recruited naive B cells. We next compiled a list of SARS-CoV-2-targeted MAbs from the literature and found that the mean SHM of 717 SARS-CoV-2-targeted antibodies is only 2.64% (Fig. 5C). Similarly, most published MAbs targeting other acute infections (such as SARS-CoV, Ebola virus, and Zika virus) also carried limited SHMs, with mean SHMs ranging from 3.42% to 7.46%. However, most MAbs identified from chronic HIV infection have a significantly higher level of SHM than MAbs against acute infections (Fig. 5C). Collectively, the activation of the SARS-CoV-2-specific antibody response is generated rapidly without extensive somatic mutations.

### SARS-CoV-2 infection induced a poly-germ line antibody response and a preferential usage of IGHJ6, leading to increased HCDR3 length.

**FIG 6:**
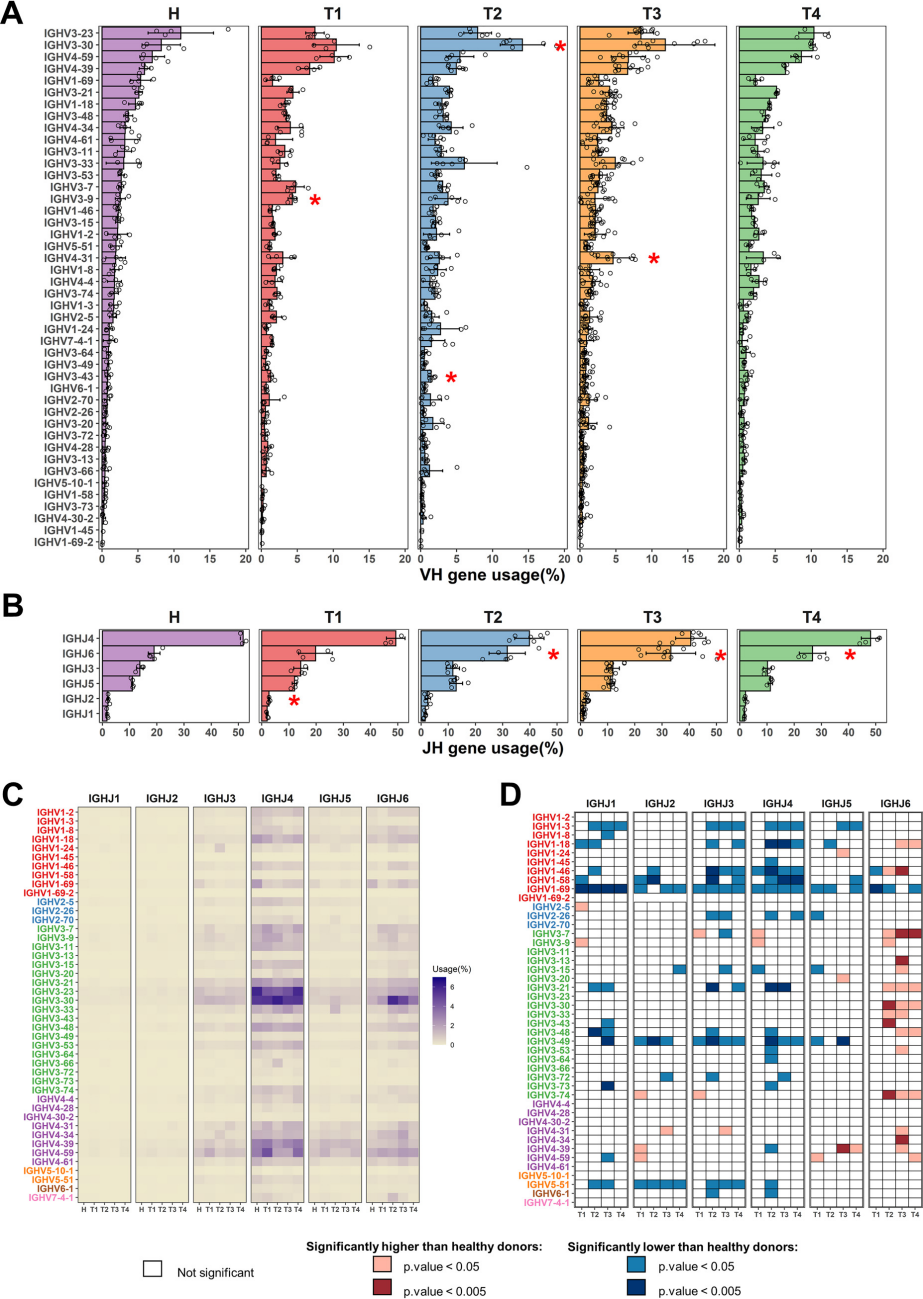
Analysis of global frequencies of IGHV/IGHJ gene usage in the IgH repertoires. (A) Global frequencies of IGHV gene usage in healthy donors and different phases of SARS-CoV-2 infection in COVID-19 patients. IGHV genes are ordered by the frequencies of healthy individuals. (B) Frequencies of IGHJ gene usage in healthy donors and different phases of SARS-CoV-2 infection in COVID-19 patients. IGHJ genes are ordered by the frequencies of healthy individuals. * in both (A) and (B) indicates significant change (*P* < 0.05) in gene usage frequency. (C) Frequencies of IGHV/IGHJ gene combination usage in healthy donors and different disease phases of COVID-19 patients. (D) Significant changes of IGHV/IGHJ gene combination usage compared with healthy individuals. White indicates no significant changes. Light and deep red indicate significantly higher, with *P* value lower than 0.05 and 0.005, respectively. Light and deep blue indicate significantly lower, with *P* value lower than 0.05 and 0.005 respectively. *P* values were calculated by *t* test.

To discover potential germ line genes associated with the response to SARS-CoV-2 infection, we first compared the frequency of IGHV genes between healthy individuals and COVID-19 patients. Consistent with previous studies (25), there is a natural frequency of IGHV gene usage in healthy people (Fig. 6A). During SARS-CoV-2 infection, the usage of several IGHV genes was significantly enriched in COVID-19 samples, including IGHV3-9, IGHV3-30, IGHV3-43, and IGHV4-31 (Fig. 6A). Consistent with the germ line gene frequency of reported SARS-CoV-2–reactive MAbs, IGHV3-30 was most frequently found in the IgH repertoire, especially in T2 (Fig. 6A and 5D). In fact, the IGHV3-30 germ line gene is commonly enlisted during different viral infections, including SARS-CoV, Ebola virus, Zika virus, and HIV-1 (13, 14, 17, 25–69). Certain IGHV germ line genes appeared to be preferentially used in response to a given virus (Fig. 5D). Notably, we found a preferential usage of IGHJ6 in the repertoires of T2, T3, and T4 (Fig. 6B). Analysis of published literatures revealed that IGHJ6 is also preferentially used in neutralizing antibodies for the Ebola virus and HIV-1 (Fig. 5E).

To provide a more detailed landscape of germ line gene expression, we further determined the IGHV/IGHJ-combination usage. The IGHV/IGHJ-combination usage showed a significantly different landscape in COVID-19 patients compared to healthy donors (Fig. 6C and D). A total of 32 unique IGHV/IGHJ combinations were found to have significantly higher expression in COIVD-19 patients than in healthy donors (Fig. 6D). Among these 32 increased IGHV/IGHJ combinations, the usage of IGHV1-18/IGHJ6, IGHV1-46/IGHJ6, IGHV3-7/IGHJ6, IGHV3-21/IGHJ6, IGHV3-30/IGHJ6, IGHV3-33/IGHJ6, IGHV3-48/IGHJ6, IGHV3-74/IGHJ6, and IGHV4-39/IGHJ5 increased in at least two time points (Fig. 6D). Intriguingly, these increased IGHV/IGHJ combinations commonly involve the IGHJ6 germ line gene. In fact, 17 of 44 IGHV genes combined with IGHJ6 showed elevated usage in at least one time point, suggesting that the IGHJ6 gene was dramatically engaged by SARS-CoV-2 infection. Overall, a subset of germ line genes was preferentially enlisted during SARS-CoV-2 infection.

**FIG 7:**
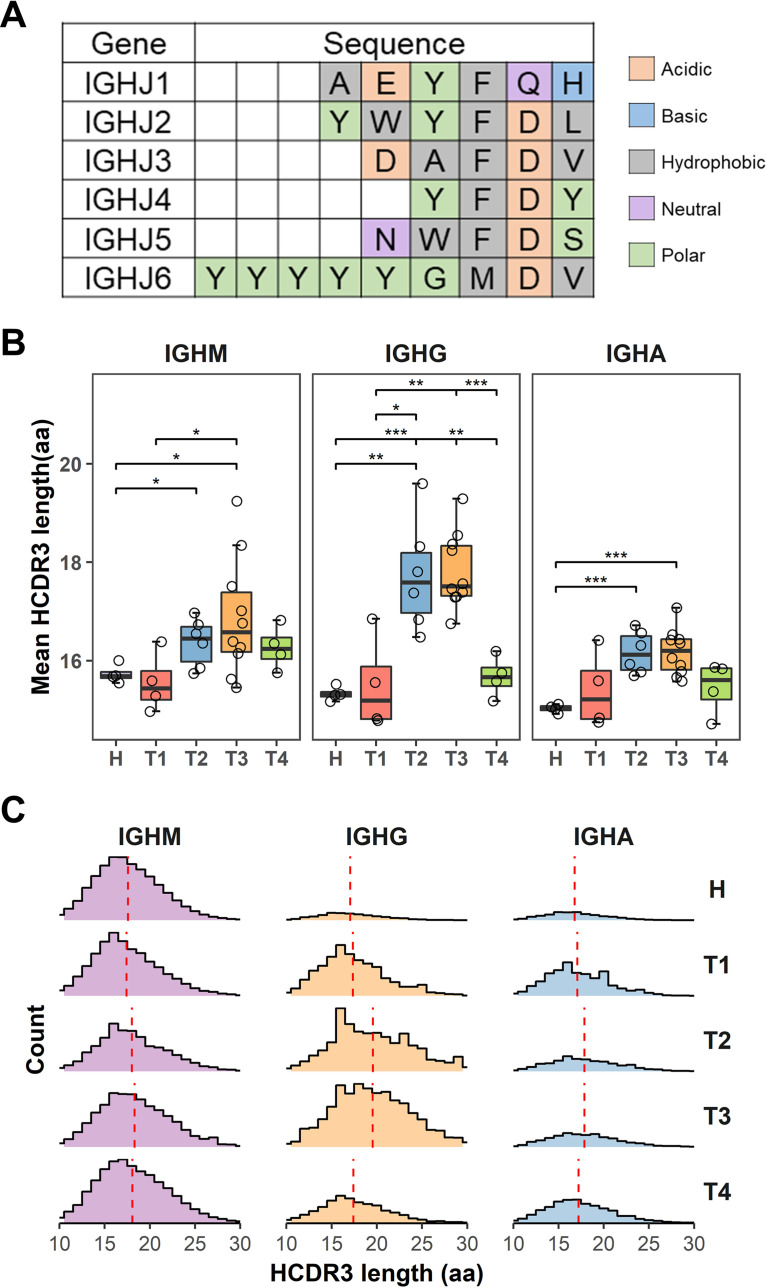
Analysis of HCDR3 length in IgH repertoires. (A) Amino acid sequences of six HJ germ line genes. (B) Mean HCDR3 length of IgH repertoire for each isotype were compared among different disease phases. (C) Count distribution of HCDR3 length for each isotype in different disease phases. The vertical red-dotted line represents mean HCDR3 length. *P* values were calculated by *t* test; *, *P* < 0.05; **, *P* < 0.01; ***, *P* < 0.001.

Given that IGHJ6 has 3 to 5 more amino acids than the other five IGHJ genes (Fig. 7A), it is likely that the increased usage of IGHJ6 contributes to a longer HCDR3 length. Indeed, we found that the mean HCDR3 length in COVID-19 patients increased dramatically during the second week after symptom onset, but decreased at 3 months after recovery (Fig. 7B and C). Notably, the HCDR3 length of the IgG antibodies increased by 2 to 3 amino acids at the T2 and T3 time points following SARS-CoV-2 infection. The HCDR3 length of the IgM and IgA also had a slight increase at the T2 and T3 time points (Fig. 7B). Therefore, the selective usage of the IGHJ6 may contribute to the increased HCDR3 length in IgG repertoires during the T2 and T3 time points. At the T4 time point, the usage of IGHJ6 and the length of HCDR3 returned to nearly the same level as the uninfected healthy people (Fig. 6C and D; Fig. 7C).

### Convergent SARS-CoV-2-specific antibodies are commonly present among COVID-19 patients.

**FIG 8:**
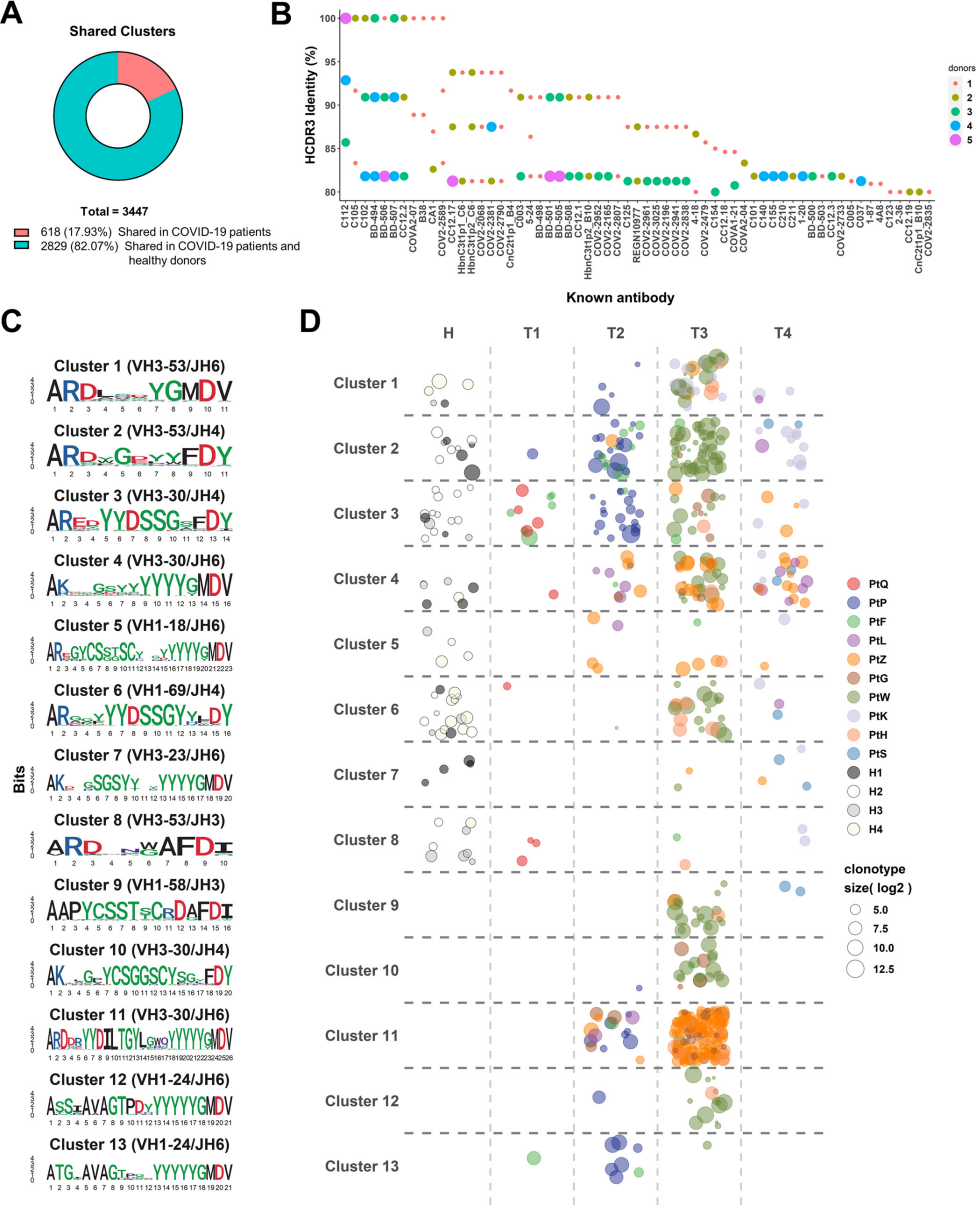
Identification of shared clonotype clusters among COVID-19 patients. (A) The number of shared clusters in COVID-19 patients or in both COVID-19 patients and healthy individuals. (B) Points represent clonotypes from repertoires of COVID-19 patients that are more than 80% similar to known neutralizing antibodies in HCDR3 amino acid sequences. Size of point indicates the number of patients that share the given antibody. (C) Sequence logos for HCDR3 amino acid sequences of the 13 shared clusters that all contain known neutralizing antibody sequences. Height of letter reflects conservation of that locus. (D) Distribution of all clonotypes of 13 shared clusters in healthy state and different phases of SARS-CoV-2 infection. Color of point is assigned by individuals. Size of point indicates the extent to which that clonotype has expanded.

To investigate the potential convergent antibody response elicited by SARS-CoV-2 infection, we performed a shared cluster analysis. Here, we defined an antibody cluster by the HCDR3 amino acid sequence identity of at least 80% with the same inferred IGHV and IGHJ genes, and a public cluster that was shared among at least three COVID-19 patients. A total of 3,447 shared clusters were identified from 10 COVID-19 donors (Fig. 8A). Among them, 2,829 (82.07%) shared clusters were also found in healthy donors, suggesting the high prevalence of shared antibodies among human naive B cell repertoires.

**FIG 9:**
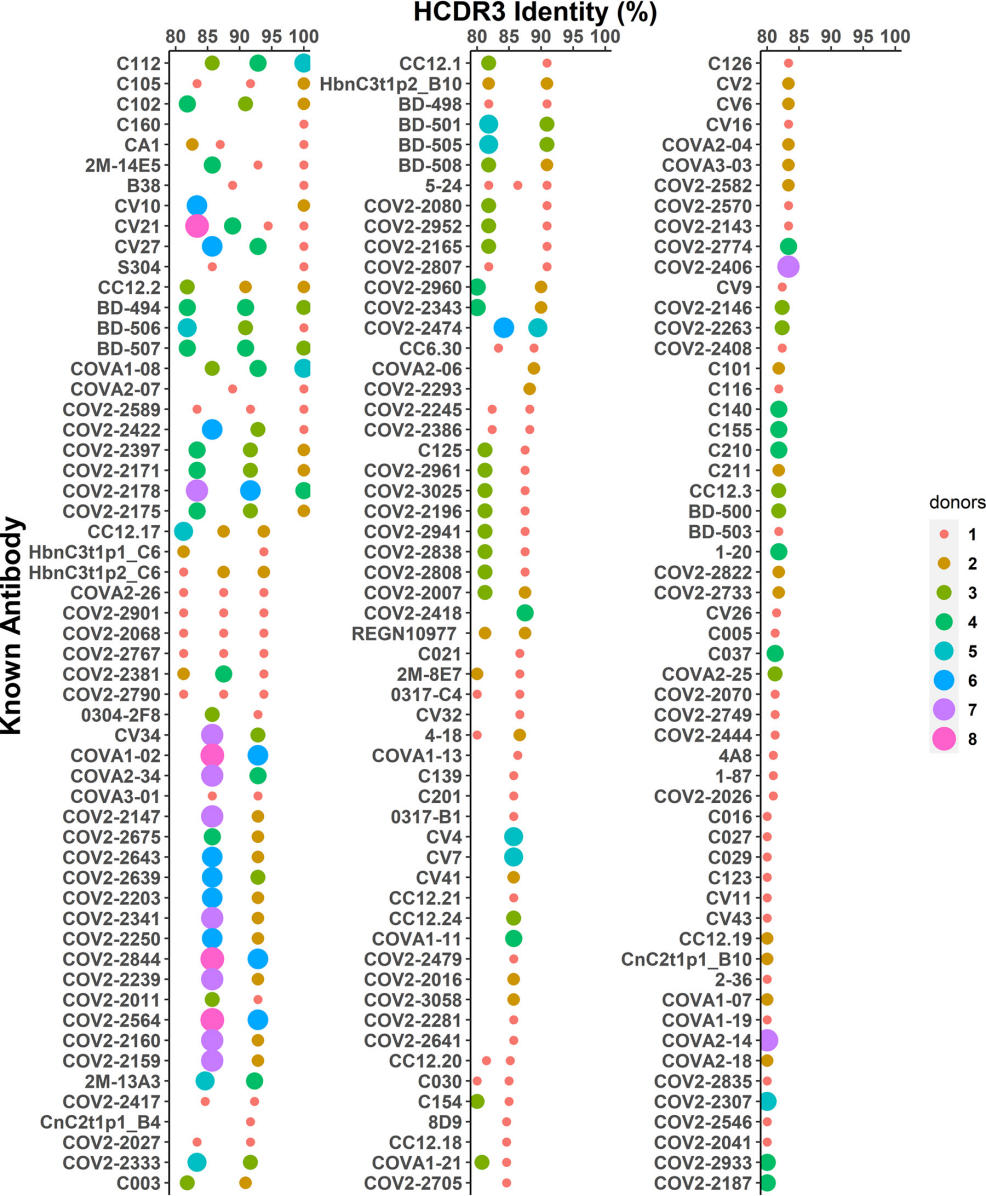
Searching similar IgH sequences in the repertoires for the known SARS-CoV-2-specific antibody sequences. Number of donors (size of points) with antibody heavy chain sequences with same V gene, same J gene, same HCDR3 length, and HCDR3 identity (*x* axis) of at least 80% to a set of known SARS-Cov-2-specific antibodies (*y* axis).

**FIG 10:**
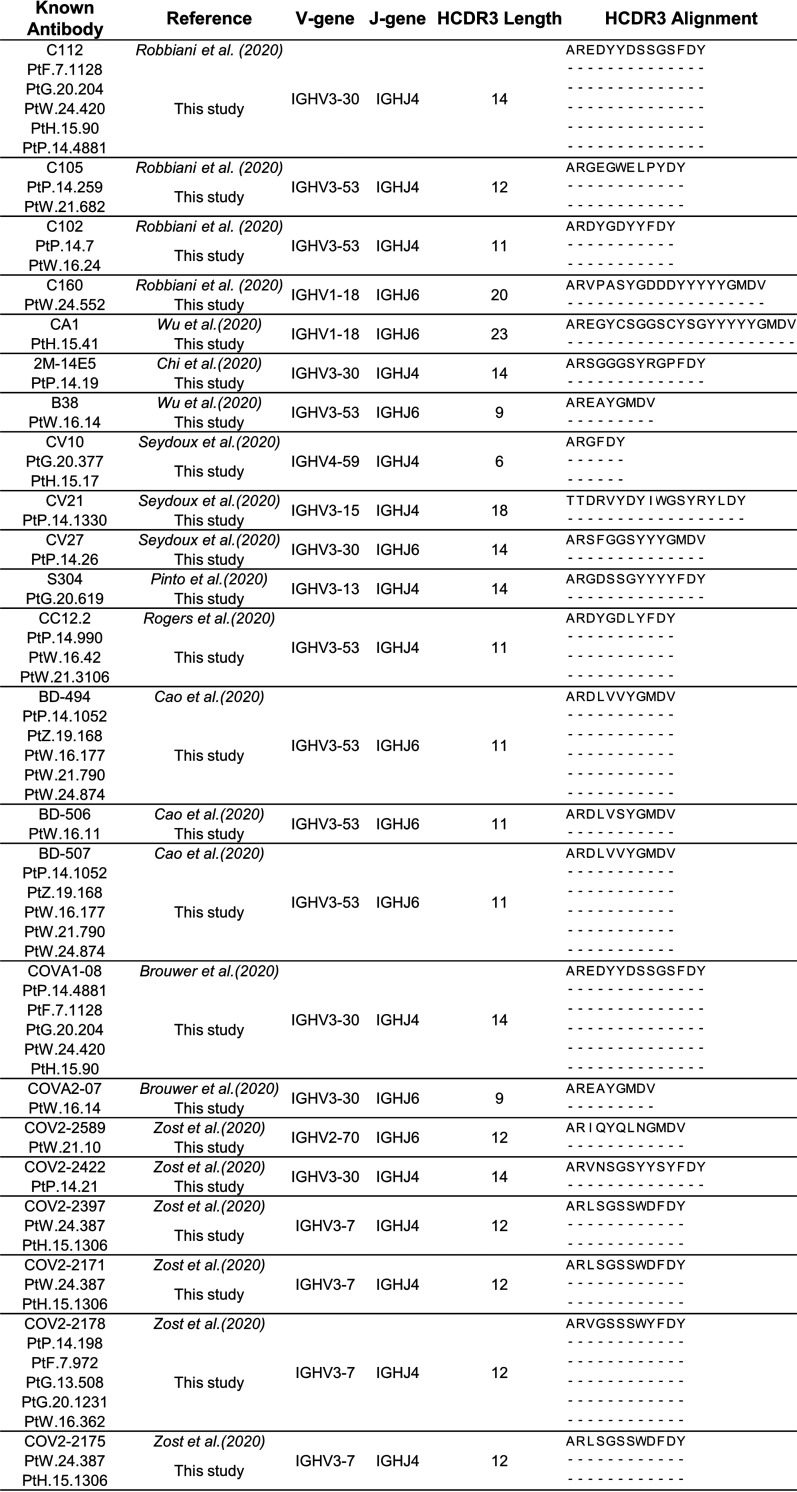
Characterization of antibodies exactly matched with 23 known SARS-CoV-2-specific antibodies. IgH profile of 23 reported SARS-CoV-2-specific antibodies with identical HCDR3 amino acids sequences in the cohorts.

To verify whether these shared clusters are SARS-CoV-2–reactive, we first performed a sequence comparison of 774 published SARS-CoV-2-specific MAbs with our IgH repertoires. One hundred sixty-eight published MAbs were found to have similar IgH sequences with the same IGHV genes and at least 80% HCDR3 amino acid sequence identity in our repertoires (Fig. 9). Among these MAbs, 61 antibodies can neutralize SARS-CoV-2 (Fig. 8B). Notably, the exact same HCDR3 of 23 reported MAbs, which were derived from 8 IGHV genes, could be identified in our repertoires. This observation indicated the convergent evolution of SARS-CoV-2-specific antibodies in the cohorts (Fig. 10). Among these 61 neutralizing MAbs, 40 can be matched to 13 shared clusters, which are highly similar to published neutralizing MAbs, including CC12.1, CC12.3, C102, REGN10977, and 4A8 (5, 6, 70, 71) (Fig. 8B and C; Table S1). Four of 13 shared clusters were derived from the IGHV3-30 germ line gene, and 3 of 13 shared clusters were derived from the IGHV3-53 germ line gene (Fig. 8C). Interestingly, 8 of 13 shared clusters were also present in healthy people's repertoires but at a much lower level of abundance (Fig. 8D). The dynamic of these 13 SARS-CoV-2-specific shared clusters echoes the dynamics of serum titers, isotype proportion, and CSR events. Some of them appeared in the second week after symptom onset, but most peaked in the third week after symptom onset (T3). Notably, although the overall abundance decreased, 9 of them were still present 3 months after recovery, demonstrating the persistence of SARS-CoV-2–responsive B cells in the peripheral blood (Fig. 8D).

## DISCUSSION

Understanding the human antibody response to SARS-CoV-2 infection has great implications for managing the COVID-19 pandemic. Mining the antibody repertoire provides a valuable perspective on how the antibodies respond to viral infection and gives insights into vaccine design and evaluation. Our study provides a global visualization of the antibody landscape in COVID-19 patients. First, patients with COVID-19 showed a transient IgA increase and steady IgG activation in antibody transcripts and serological titers. Second, a holistic view of the kinetics of antibody response showed a rapid clonal expansion and differentiation in the first 3 weeks after symptom onset, characterized by dominant clones and low diversity. Our results revealed that the proliferating or transcriptionally activated B cells were mainly from the naive B cell population instead of the antigen-experienced part, as evidenced by the sharply increased proportion of low-SHM IgH sequences upon SARS-CoV-2 infection. Third, a convergent antibody response that engaged multiple clonotypes was found in COVID-19 patients. The major shared clusters with convergent IGHV usages were clones identified by matching reported receptor-binding domain- (RBD)-targeted MAbs with no or low SHM. These clonotypes flourished during acute infection, and most of them were still detectable 3 months after discharge from hospitalization. Some clonotypes could also be found in healthy donors, suggesting that precoded antibodies are poised for rapid response to SARS-CoV-2 infection in some people, which may help them confer better early defense.

Analysis of antibody repertoires of COVID-19 patients revealed a burst of IgG and IgA clones with low SHM in T2 and T3, indicating SARS-CoV-2-specific IgG and IgA clones derived from naive B cells in these time points. This finding is in line with our analysis that the mean SHM rate of 774 published SARS-CoV-2-targeted antibodies is only 2.64%. This appears to be the lowest SHM rate among all of the other antibodies targeting other acute infections. The published MAbs against other infections such as SARS-CoV, Ebola virus, and Zika virus also have limited SHM, with a mean SHM ranging from 3.42% to 7.46%. In contrast, most antibodies induced by chronic HIV infection have a significantly higher level of mean SHM (14.53%) than antibodies against acute infections (Fig. 5C). Collectively, the activation of the SARS-CoV-2-specific antibody response is generated rapidly without extensive somatic mutations.

The global immunoglobulin CSR events in the repertoires could be visualized at the transcriptomic level. Other investigators and we have observed early and extensive CSR to IgA with no or low SHM in the first week after infection, highlighting the effect of IgA antibodies in the respiratory mucosa to combat virus infection (7, 21). Of note, IGHA switching to IGHG1/IGHG2 events persisted for 3 months after virus clearance. A recent report also showed that IgM and IgG antibody titers against the spike protein decreased over time while IgA was less affected 6 months after infection (72). Thus, IgA antibodies may provide an important protection mechanism against SARS-CoV-2 in the respiratory tract.

Preferential germ line gene usages were observed in the antibody repertoires of Zika virus-infected patients, H7N9 avian influenza-infected patients, and rVSV-ZEBOV immunized individuals (15, 25, 73). We found a preferential usage of IGHV3-9, IGHV3-30, IGHV3-43, IGHV4-31, and IGHJ6 germ line genes in COVID-19 patients. Furthermore, 17 IGHV genes combined with IGHJ6 increased in COVID-19 samples, highlighting the critical role of IGHJ6 in the SARS-COV-2-elicited antibody responses. This observation coincided with a recent study showing a salient preference for IGHV3 genes rearranged with IGHJ6 in COVID-19 repertoires (74). These germ line genes were also frequently seen in SARS-CoV-2-specific MAbs (3). Unveiling the structural mechanism of these IGHJ6-preferred antibodies binding to spike protein may guide the vaccine design. Vaccines aimed at eliciting a specific subset of antibodies may offer rapid and effective protection.

Previous studies have found the induction of shared antibodies in some virus infections, such as EBOV, HIV-1, influenza, and dengue virus (15, 75–77). Recent studies also found shared clusters in COVID-19 patients (21, 78, 79). However, it is not clear whether these shared clusters are specifically elicited by SARS-CoV-2 infection. If they are specific, it also remains to be elucidated how long these specific shared clusters will last and how their expression levels change over time. This study identified 13 shared clusters that were highly relevant with known neutralizing antibodies isolated from COVID-19 patients. Recent reports showed that IGHV3-53 and IGHV3-30 encode convergent antibodies targeting the RBD of spike protein (5, 72, 80–82). These results supported that SARS-CoV-2 induced convergent antibody response across different patients, which can be identified by querying antibody sequencing repertoires. Furthermore, we tracked the dynamic of the shared SARS-CoV-2-specific clusters and their changes over time. The shared SARS-CoV-2-specific clusters increased at 2–3 weeks after symptom onset, suggesting they take part in the initial infection control. Notably, a significant number of shared SARS-CoV-2-responsive clusters were still present in the antibody repertoire at 3 months after patients were virus-free. Although this finding supports the view that SARS-CoV-2 infection induces the generation of long-lived plasma cells or memory B cells at the antibody repertoire level, it remains unknown if the magnitude of these B populations effectively prevents secondary infections. One limitation of this analysis is that only four patients were followed up to 3 months. The dynamic of antibody responses induced by SARS-CoV-2 infection requires more systematic and in-depth research on a larger cohort over a longer period. Antibodies identified from these shared spike-specific clusters possess binding and neutralizing activities, as demonstrated in our recent report that synthesized a repertoire-deduced IGHV3-53-encoded heavy chain paired with a common IGKV1-9 light chain were successfully expressed in transfected HEK293 cells and showed RBD binding and virus-neutralizing activities (82).

Another interesting finding is that 8 shared SARS-CoV-2-specific clusters were found at a low level of abundance in the repertoires of several healthy individuals. Our finding coincided with a report that SARS-CoV-2-specific B cells can be identified among healthy individuals and cancer patients without prior SARS-CoV-2 exposure (78, 83). These findings highlight the existence of antibody precursor sequences targeting SARS-CoV-2 in human antibody repertoires. Certain germ line genes are preferentially enlisted to generate convergent SARS-CoV-2-specific antibodies in different people. These rapidly generated antibodies are characterized by no or minimal SHM, likely without the need to undergo affinity maturation process in the germinal center. Therefore, we propose that the human antibody repertoire is poised to prompt rapid responses to infections of SARS-CoV-2 and likely some other pathogens.

## MATERIALS AND METHODS

### Serum binding assay.

The 96-well plates were coated with SARS-CoV-2 Spike (S) protein (Sino biological) at 1 μg/mL in DPBS overnight, and then blocked with 5% nonfat milk. Serum samples were used with a 2-fold serial dilution starting at 1:50 in 1× PBS, and 100 μL of each sample was applied to coated ELISA plates and incubated for 2 h at 37°C. Plates were then washed, and each well was incubated in 100 μL with HRP-labeled antihuman IgM (Sigma-Aldrich, MI, USA), IgG (Beyotime), or IgA (Abcam), diluted to 1:2,000, 1:5,000, and 1:8,000 in 5% nonfat milk in 1× PBS at 37°C for 1 h. Following extensive washing, plates were developed with TMB/E substrate (Merck Millipore, MA, USA) at room temperature for 15 min. Finally, the reaction was stopped with 1 M H2SO4, and the optical density values at 450 nm were read. Negative serum control was run each time the assay was performed. The cutoff value for seropositive samples was set as the mean value at OD450 for the five negative serum samples plus three standard deviations.

### IgH library preparation.

This study was approved by the Ethics Committee of Guangzhou Eighth People's Hospital (202012145). PBMCs were isolated from blood samples, and total RNA was extracted using TRIzol LS reagent according to the manufacturer's protocol (Life Technologies). ARMS-PCR using multiplex primers (iRepertoire, Inc, Huntsville, AL, USA) was performed to amplify the IgH repertoire sequences as described previously (18). Briefly, multiplex primers covering the human IGHV genes (forward primers) and constant region primers (reverse primers) were designed. The forward primers Fi (forward-in) and reverse primers Ri (reverse-in) also included Illumina paired-end sequencing communal primers B and A, respectively (Illumina, San Diego, CA, USA). Unique barcodes introduced in the first round by the constant region primers were used to distinguish the samples.

### Sequencing and barcode filtering.

After gel purification using a QIAquick gel extraction kit (Cat. No. 28704; Qiagen), the product was pooled and sequenced on Illumina NovaSeq 6000 with paired-end 250 bp read mode (Novogene, China). Raw sequencing results were filtered for base quality (3′ ends of read sequences with quality scores over 20 were retained) using Trimmomatic v0.36 (84). After filtering, the paired-end reads were separated based on the unique barcodes at the 5′ end of the reads. The separated reads were merged using FLASH v1.2.11 (85) if their overlapping regions were more than 30 bp. Finally, the whole-length antibody sequences with 300 bp–470 bp were annotated within the V(D)J germ line genes using MIXCR v3.0.3 (86), and the reference V(D)J sequences were downloaded from the IMGT database (http://www.imgt.org/).

### Clonotype analysis.

Clustering highly similar antibody sequences is an effective way to mitigate PCR and sequencing errors (87). Therefore, we defined antibody clonotype by the same V gene, the same J gene, the same HCDR3 length, and identical HCDR3 amino acid sequences. The reads in each unique clonotype were considered as the clonotype size. Clonotypes containing only one read were considered to result from sequencing bias and were removed before the subsequent analysis. In addition, clonotype size was normalized to the number of reads for a clonotype per 10,000 reads in each IgH repertoire to modify the bias of sequencing depth between different samples.

### Isotype frequencies, somatic hypermutation, HCDR3 length, and IGHV/IGHJ gene usage.

We calculated isotype usage, mean somatic hypermutation, HCDR3 length, and IGHV/IGHJ gene usage by normalized clonotypes to mitigate the potential sequencing biases from differential RNA per cell.

### Clonal index analysis.

The clonal index analysis was performed as described by Bashford-Rogers et al. (24). Clonal expansion index was calculated with all of the clones ordered by the number of reads in each clone, and a clonal diversification index was calculated with all clusters ordered by the number of unique clonotypes in each cluster. In addition, the index of specific cell types or isotypes was calculated within the subsets of B cells.

The equation for calculating the clonal index is

Clonal index=∑i=1N(ri−(iN))N

where *r*_i_ is any object of R = {*r*_1_, *r*_2_, …, *r*_n_}, a set of the accumulative size of clones or clusters ordered from the largest to the smallest. *N* is the number of clones or clusters.

### CSR events analysis.

A B cell encoding a unique clonotype that exists in more than one isotype is reckoned to have undergone CSR from the upstream isotype to the downstream isotype along the C gene order in the Ig heavy chain. Therefore, for each isotype, there is a certain number of defined class-switched clonotypes that come from or flow to other isotypes at a certain time point. The frequency of CSR between any two isotypes is calculated as the proportion of clonotypes they share in all class-switched clonotypes of the upstream isotype. Here, we assume the CSR event is simply restricted to two isotypes despite the indirect pathway. In other words, when some clonotypes are found in three isotypes (IgX, IgY, IgZ, for example), the three possible modes of CSR event are equally considered mode (a), shown in the class-switching recombination part of the antigen-driven antibody affinity maturation section (Fig. 1A). Furthermore, the two subclasses of IgA (IgA1 and IgA2) and IgG (IgG1 and IgG2) are respectively grouped into IgA and IgG1/IgG2 due to their undistinguishable sequencing segment as a primer.

### Shared cluster analysis.

To analyze shared clusters, the unique clonotype from 14 donors (10 COVID-19 patients and 4 healthy donors) was identified. Clonotypes with at least 5 reads were used for further analysis, avoiding potential biases caused by sequencing. Here, we defined a cluster by the HCDR3 amino acid sequence identity of at least 80% and the same inferred V and J genes. In addition, if a cluster was shared among at least 3 COVID-19 patients, it was considered a shared cluster.

### Comparison of IgH-Seq data to known SARS-CoV-2 neutralizing antibodies.

A list of known SARS-CoV-2 neutralizing antibodies was curated manually from the recent studies (3–6, 26, 70, 71, 80, 81, 88–93). Requirements for collecting sequences were (i) definitely inferred V gene and complete HCDR3 sequences and (ii) the nucleotide sequence of the antibody heavy chain. The nucleotide sequence was processed via MiXCR v3.0.3 (86). Highly similar clonotypes were amino acid sequence-based at an 80% HCDR3 identity threshold, with the same inferred V(J) genes found in known SARS-CoV-2 neutralizing antibodies.

### Statistics.

Statistical analysis was performed in R. To characterize changes among healthy individuals (H) and COVID-19 patients in different infection periods (T1–T4), Student's *t-*test was performed as mentioned in figure legends. To assess the correlation between the proportion of low-SHM antibody (SHM rate < 2%) and ELISA titer for IgM, IgG, and IgA binding to SARS-CoV-2 spike protein and SARS-CoV-2 nucleocapsid protein, Pearson correlation analysis was performed, and linear models were fitted. In all of the bar plots, mean ± standard deviation was depicted. *P* values were considered to be significant when < 0.05.

### Data availability.

All raw IgH repertoire data used in this study have been deposited at the National Genomics Data Center (https://bigd.big.ac.cn/) under accession number PRJCA007067.
