# Supplementary material for: Analysis of B Cell Receptor Repertoires Reveals Key Signatures of the Systemic B Cell Response after SARS-CoV-2 Infection
Source: J Virol. 2022 Feb 23;96(4):e01600-21. doi: 10.1128/jvi.01600-21 (PMC8865482; doi:10.1128/jvi.01600-21)
Supplement: Supplemental file 1 — Table S1. Download jvi.01600-21-s0001.pdf, PDF file, 0.03 MB [file jvi.01600-21-s0001.pdf]

Table S1. Clusters of SARS-CoV-2 spike-specific antibodies.

Cluster	mAb ID	IGHV	IGHJ	HCDR3	HCDR3 length	Reference
Cluster1	BD-494	IGHV3-53	IGHJ6	ARDLVVYGMDV	11	Cao et al. (2020) (1)
	BD-500	IGHV3-53	IGHJ6	ARDAMSYGMDV		
	BD-501	IGHV3-53	IGHJ6	ARDRVVYGMDV		
	BD-503	IGHV3-53	IGHJ6	ARDAAVYGIDV		
	BD-505	IGHV3-53	IGHJ6	ARDRVVYGMDV		
	BD-506	IGHV3-53	IGHJ6	ARDLVSYGMDV		
	BD-507	IGHV3-53	IGHJ6	ARDLVVYGMDV		
	BD-508	IGHV3-53	IGHJ6	ARDAQNYGMDV		
	C140	IGHV3-53	IGHJ6	ARDLYYYGMDV		Robbiani et al. (2020) (2)
	C210	IGHV3-53	IGHJ6	ARDLMAYGMDV		
	COV2-2952	IGHV3-53	IGHJ6	ARDLVTYGLDV		Zost et al.(2020) (3)
	COV2-2165	IGHV3-53	IGHJ6	ARDLVTYGLDV		
	CC12.1	IGHV3-53	IGHJ6	ARDLDVYGLDV		Rogers et al.(2020) (4)
	1-20	IGHV3-53	IGHJ6	ARDLFYYGMDV		Liu et al.(2020) (5)
Cluster2	C003	IGHV3-53	IGHJ4	ARDYGDFYFDY	11	Robbiani et al. (2020) (2)
	C101	IGHV3-53	IGHJ4	VRDYGDFYFDY		
	C102	IGHV3-53	IGHJ4	ARDYGDYYFDY		
	C155	IGHV3-53	IGHJ4	ARDFGEFYFDY		
	CC12.2	IGHV3-53	IGHJ4	ARDYGDLYFDY		Rogers et al.(2020) (4)
	CC12.3	IGHV3-53	IGHJ4	ARDFGDFYFDY		
Cluster3	C112	IGHV3-30	IGHJ4	AREDYYDSSGSFDY	14	Robbiani et al. (2020) (2)
Cluster4	CC12.17	IGHV3-30	IGHJ6	AKSSGSYYYYYYGMDV	16	Rogers et al.(2020) (4)
Cluster5	CA1	IGHV1-18	IGHJ6	AREGYCSGGSCYSGYYYYYGMDV	23	Shi et al. (2020) (6)
Cluster6	REGN10977	IGHV1-69	IGHJ4	ARTPFYYDSSGYYLDY	16	Hansen et al. (2020) (7)
Cluster7	CC12.19	IGHV3-23	IGHJ6	AKGSGSGSYPNYYYYYGMDV	20	Rogers et al.(2020) (4)
Cluster8	C123	IGHV3-53	IGHJ3	ARDLSAAFDI	10	Robbiani et al. (2020) (2)
Cluster9	C037	IGHV1-58	IGHJ3	AAPYCSGGSCNDAFDI	16	Robbiani et al. (2020) (2)
	C125	IGHV1-58	IGHJ3	AAPYCSGGSCSDAFDI		
	HbnC3t1p1_C6	IGHV1-58	IGHJ3	AAPHCSSTICYDGFDI		Kreer et al.(2020) (8)
	HbnC3t1p2_C6	IGHV1-58	IGHJ3	AAPYCSSTRCYDAFDI		
	COV2-2961	IGHV1-58	IGHJ3	AAPYCSSISCNDGFDI		Zost et al.(2020) (3)
	COV2-3025	IGHV1-58	IGHJ3	AAPYCSSISCNDGFDI		
	COV2-2196	IGHV1-58	IGHJ3	AAPYCSSISCNDGFDI		
	COV2-2381	IGHV1-58	IGHJ3	AAPYCSRTSCHDAFDI		

	COV2-2941	IGHV1-58	IGHJ3	AAPYCSSISCNDGFDI		
	COV2-2838	IGHV1-58	IGHJ3	AAPYCSSISCNDGFDI		
Cluster10	C154	IGHV3-30	IGHJ4	AKQAGPYCSGGSCYSAPFDY	20	Robbiani et al. (2020) (2)
Cluster11	COVA1-21	IGHV3-30	IGHJ6	ARDSEYYDILTGYLAPTHYYYYYMDV	26	Brouwer et al.(2020) (9)
Cluster12	4A8	IGHV1-24	IGHJ6	ATSTAVAGTPDLFDYYYGMDV	21	Chi et al.(2020) (10)
Cluster13	1-87	IGHV1-24	IGHJ6	ATGIAVIGPPPSTYYYYGMDV	21	Liu et al.(2020) (5)

Reference

1. Cao YL, Su B, Guo XH, Sun WJ, Deng YQ, Bao LL, Zhu QY, Zhang X, Zheng YH, Geng CY, Chai XR, He RS, Li XF, Lv Q, Zhu H, Deng W, Xu YF, Wang YJ, Qiao LX, Tan YF, Song LY, Wang GP, Du XX, Gao N, Liu JN, Xiao JY, Su XD, Du ZM, Feng YM, Qin C, Qin CF, Jin RH, Xie XS. 2020. Potent Neutralizing Antibodies against SARS-CoV-2 Identified by High-Throughput Single-Cell Sequencing of Convalescent Patients' B Cells. Cell 182:73-+.
2. Robbiani DF, Gaebler C, Muecksch F, Lorenzi JCC, Wang Z, Cho A, Agudelo M, Barnes CO, Gazumyan A, Finkin S, Hagglof T, Oliveira TY, Viant C, Hurley A, Hoffmann HH, Millard KG, Kost RG, Cipolla M, Gordon K, Bianchini F, Chen ST, Ramos V, Patel R, Dizon J, Shimeliovich I, Mendoza P, Hartweger H, Nogueira L, Pack M, Horowitz J, Schmidt F, Weisblum Y, Michailidis E, Ashbrook AW, Waltari E, Pak JE, Huey-Tubman KE, Koranda N, Hoffman PR, West AP, Jr., Rice CM, Hatziioannou T, Bjorkman PJ, Bieniasz PD, Caskey M, Nussenzweig MC. 2020. Convergent antibody responses to SARS-CoV-2 in convalescent individuals. Nature 584:437-442.
3. Zost SJ, Gilchuk P, Chen RE, Case JB, Reidy JX, Trivette A, Nargi RS, Sutton RE, Suryadevara N, Chen EC, Binshtein E, Shrihari S, Ostrowski M, Chu HY, Didier JE, MacRenaris KW, Jones T, Day S, Myers L, Eun-Hyung Lee F, Nguyen DC, Sanz I, Martinez DR, Rothlauf PW, Bloyet LM, Whelan SPJ, Baric RS, Thackray LB, Diamond MS, Carnahan RH, Crowe JE, Jr. 2020. Rapid isolation and profiling of a diverse panel of human monoclonal antibodies targeting the SARS-CoV-2 spike protein. Nat Med 26:1422-1427.
4. Rogers TF, Zhao F, Huang D, Beutler N, Burns A, He WT, Limbo O, Smith C, Song G, Woehl J, Yang L, Abbott RK, Callaghan S, Garcia E, Hurtado J, Parren M, Peng L, Ramirez S, Ricketts J, Ricciardi MJ, Rawlings SA, Wu NC, Yuan M, Smith DM, Nemazee D, Teijaro JR, Voss JE, Wilson IA, Andrabi R, Briney B, Landais E, Sok D, Jardine JG, Burton DR. 2020. Isolation of potent SARS-CoV-2 neutralizing antibodies and protection from disease in a small animal model. Science 369:956-963.
5. Liu L, Wang P, Nair MS, Yu J, Rapp M, Wang Q, Luo Y, Chan JF, Sahi V, Figueroa A, Guo XV, Cerutti G, Bimela J, Gorman J, Zhou T, Chen Z, Yuen KY, Kwong PD, Sodroski JG, Yin MT, Sheng Z, Huang Y, Shapiro L, Ho DD. 2020. Potent neutralizing antibodies against multiple epitopes on SARS-CoV-2 spike. Nature 584:450-456.
6. Shi R, Shan C, Duan XM, Chen ZH, Liu PP, Song JW, Song T, Bi XS, Han C, Wu LA, Gao G, Hu X, Zhang YA, Tong Z, Huang WJ, Liu WJ, Wu GZ, Zhang B, Wang L, Qi

JX, Feng H, Wang FS, Wang QH, Gao GF, Yuan ZM, Yan JH. 2020. A human neutralizing antibody targets the receptor-binding site of SARS-CoV-2. Nature doi:10.1038/s41586-020-2381-y.

7. Hansen J, Baum A, Pascal KE, Russo V, Giordano S, Wloga E, Fulton BO, Yan Y, Koon K, Patel K, Chung KM, Hermann A, Ullman E, Cruz J, Rafique A, Huang T, Fairhurst J, Libertiny C, Malbec M, Lee WY, Welsh R, Farr G, Pennington S, Deshpande D, Cheng J, Watty A, Bouffard P, Babb R, Levenkova N, Chen C, Zhang B, Romero Hernandez A, Saotome K, Zhou Y, Franklin M, Sivapalasingam S, Lye DC, Weston S, Logue J, Haupt R, Frieman M, Chen G, Olson W, Murphy AJ, Stahl N, Yancopoulos GD, Kyratsous CA. 2020. Studies in humanized mice and convalescent humans yield a SARS-CoV-2 antibody cocktail. Science 369:1010-1014.
8. Kreer C, Zehner M, Weber T, Ercanoglu MS, Gieselmann L, Rohde C, Halwe S, Korenkov M, Schommers P, Vanshylla K, Di Cristanziano V, Janicki H, Brinker R, Ashurov A, Krahling V, Kupke A, Cohen-Dvashi H, Koch M, Eckert JM, Lederer S, Pfeifer N, Wolf T, Vehreschild M, Wendtner C, Diskin R, Gruell H, Becker S, Klein F. 2020. Longitudinal Isolation of Potent Near-Germline SARS-CoV-2-Neutralizing Antibodies from COVID-19 Patients. Cell 182:843-854 e12.
9. Brouwer PJM, Caniels TG, van der Straten K, Snitselaar JL, Aldon Y, Bangaru S, Torres JL, Okba NMA, Claireaux M, Kerster G, Bentlage AEH, van Haaren MM, Guerra D, Burger JA, Schermer EE, Verheul KD, van der Velde N, van der Kooi A, van Schooten J, van Breemen MJ, Bijl TPL, Sliepen K, Aartse A, Derking R, Bontjer I, Kootstra NA, Wiersinga WJ, Vidarsson G, Haagmans BL, Ward AB, de Bree GJ, Sanders RW, van Gils MJ. 2020. Potent neutralizing antibodies from COVID-19 patients define multiple targets of vulnerability. Science 369:643-650.
10. Chi X, Yan R, Zhang J, Zhang G, Zhang Y, Hao M, Zhang Z, Fan P, Dong Y, Yang Y, Chen Z, Guo Y, Zhang J, Li Y, Song X, Chen Y, Xia L, Fu L, Hou L, Xu J, Yu C, Li J, Zhou Q, Chen W. 2020. A neutralizing human antibody binds to the N-terminal domain of the Spike protein of SARS-CoV-2. Science 369:650-655.
